# The Results of Resection of Intestine

**Published:** 1900-02-03

**Authors:** 


### THE RESULTS OF RESECTION OF INTESTINE.

In many modern abdominal operations the amount of bowel which has to be excised is so considerable that it becomes a very important matter to ascertain to what extent the human intestine may be removed without entailing, if not actual death from inanition, at least serious interference with the function of absorption. Various experiments have been made with the object of deciding this point. As the result of the observations made by Senn he came to the conclusion that the excision of more than one-third of the whole length of the small intestine was an operation dangerous to life, productive of marasmus, and in this way fatal sooner or later. Trzebicky assigned much wider limits for the intestinal resections permissible in dogs, and he has also shown that much depends upon which part of the small intestine is removed, excision of the beginning of the jejunum being much more serious in its effect than removal of the part nearer the ilio-cæcal valve. There being at present so much divergence of opinion as to the amount of intestine which can be removed with safety, Professor Carl Schlatter[1] of Zurich, with the object of helping to clear up the doubt, has lately made a series of careful observations of the process of digestion in a case under his care in which, in consequence of a wound of the abdomen it had been necessary to excise a length of 192 centimetres of small intestine which was found protruding. The whole of the portion removed belonged to the ileum. Two months after the operation the patient was discharged recovered. He had gained 11 kilogrammes during the preceding month, his appetite was hearty, his bowels acted regularly almost every day, his strength was good, and he presented the appearance of exuberant health. Careful and elaborate estimations of the amounts of nitrogen and fat taken and excreted showed that while the loss of nitrogen in the fæces was at the upper limit of the normal that of the fat was deficient, thus indicating that the assimilation of fat was imperfect. If, then, merely clinical observations had been relied upon it might have been said that the large loss of intestine which had occurred in this case had made no difference, and had been in no way dangerous to the organism so far as digestion was concerned. But an examination of the experiments made as to food assimilation taught one to be cautious in accepting the clinical evidence by itself, and the ultimate progress of the case showed that such caution was necessary, for eight months after the operation it was found that the patient, although he could do a little work in the field, worked only slowly, and with many intervals for rest. He lived principally upon broth, soup, and animal food, and was quite unable to tolerate the usual food of his class, such as bread, polenta, cheese, &c. Moreover, he had lost a little flesh—about three kilogrammes—since leaving the hospital. This result should then serve as a warning against accepting too optimistic views regarding the results of extensive resections, for it shows that, contrary to what has hitherto been the received opinion, the function of the digestive tract is seriously interfered with, even in young persons, by the resection of more than two metres of the bowel, unless precautions are taken to secure the requisite sustenance.

[1] Lancet, Jan. 27.

